# Adapting a conceptual framework to engage diverse stakeholders in genomic/precision medicine research

**DOI:** 10.1111/hex.13486

**Published:** 2022-03-30

**Authors:** Karriem S. Watson, Elizabeth G. Cohn, Alecia Fair, Usha Menon, Laura A. Szalacha, Selena M. Carpenter, Consuelo H. Wilkins

**Affiliations:** ^1^ Division of Community Health Sciences, School of Public Health, Mile Square Health Center University of Illinois Chicago Illinois USA; ^2^ Hunter‐Bellevue School of Nursing, Hunter College New York New York USA; ^3^ Department of Medicine Vanderbilt University Medical Center and the Meharry‐Vanderbilt Alliance Nashville Tennessee USA; ^4^ College of Nursing University of South Florida Tampa Florida USA; ^5^ Morsani Health College of Medicine and College of Nursing University of South Florida Tampa Florida USA; ^6^ Office of Health Equity Vanderbilt University Medical Center Nashville Tennessee USA

**Keywords:** conceptual framework, genomics, precision medicine, stakeholder engagement

## Abstract

**Introduction:**

Genomic/precision medicine offers a remarkable opportunity to improve health and address health disparities. Genomic medicine is the study of genes and their interaction with health. Precision medicine is an approach to disease prevention and treatment that considers individual variability in genes, environment and lifestyle. Conclusions from studies lacking diversity may hinder generalizability as genomic variation occurs within and between populations. Historical factors, such as medical mistrust, ethical issues related to decision making, and data sharing pose complex challenges that may further widen inequities in genomic/precision medicine if not appropriately addressed. Although few biomedical studies integrate priorities of community partners into their conceptual framework, effective implementation of genomic/precision medicine research calls for the involvement of diverse stakeholders to expand traditional unidirectional models of engagement in clinical research towards authentic bidirectional collaboration.

**Methods:**

A multipronged approach was used integrating an evidence‐based literature review and best practices in developing and evaluating the engagement of diverse stakeholders in genomic and precision medicine research. This was combined with expert consensus building to adapt a conceptual model from a community engagement framework to addressing genomics to be scalable to engagement science, which is challenging to genomic/precision medicine research.

**Results:**

The final enhanced conceptual framework is composed of four overarching dimensions now inclusive of domains in trust, exploitation, discrimination, privacy risk, stigmatization, prior harms/injustices, failure to recognize coexisting governments, intersectionality and research transformation. This conceptual framework proposes effective participant research engagement strategies for upstream relationship building, distinct from downstream recruitment strategies in which the goal is enrolment.

**Conclusion:**

To further shape the evolution of genomic/precision medicine research, it is important to leverage existing partnerships, engage participants beyond recruitment and embrace diverse perspectives.

**Patient or Public Contribution:**

In preparation of this manuscript, the perspectives of the community partners on the impact of engaging in genomic/precision medicine research beyond research participation were integrated into this conceptual framework from various guided listening sessions held in diverse communities.

## 1 INTRODUCTION

Precision medicine promises to transform health care by accounting for individual genetic variability, environment and lifestyle to deliver individualized interventions.^1^ As such, genomics is a central component of precision medicine.^2^ However, some populations may not reap any benefit from genomic/precision medicine.

Although more than 75% of the global population is of Asian or African ancestry, many studies use existing genomic databases largely comprised of individuals of European ancestry.^3^ Genomic variation occurs within and between populations; thus, conclusions from studies lacking diversity may lack generalizability and could exacerbate disparities. Racial and ethnic, as well as sociocultural and environmental diversities in research increases identification of genomic variants; more comprehensively identifies genomic data relevant to racial/ethnic groups, who often have the highest disease burden accelerating the translation of discoveries into practice.

Engaging historically underrepresented racial/ethnic minorities in genomic/precision medicine is a major challenge. There are harrowing legacies of abuses associated with research that contribute to this challenge, including commercialization of biospecimens, breaches of confidentiality, eugenics throughout history signifying scientific racism, traumatizing interpretations of data^4^ and population origin studies that violate religious beliefs and invalidate ethnic identities. Genetic/precision medicine has been associated with studies in which genetic information was used with injurious intent and to politicize medicine.^5^, ^6^ The complexities of genomics, use of DNA as criminal evidence in justice systems that unjustly harm minorities, and disparities in privacy risks^7^ continue to contribute to distrust in genomic research.^8^, ^9^

The scope of genomic/precision medicine has shifted from disease‐specific studies to large‐scale cohorts intended to address health disparities.^10^ The norm in genomic/precision medicine is unidirectional recruitment. Although engagement has been shown to increase minority recruitment, engagement goals must differentiate from recruitment goals, which focus on study enrolment. There must be deliberate bidirectional engagement between researchers and racial/ethnic minorities. This relationship should focus on understanding diverse communities' perspectives to enhance study design, more precisely assess genetic, social and environmental risks to guide interpretation of findings, and identify barriers and facilitators to translating discoveries into practice.^11^

Diverse communities have been engaged in health research for decades using community‐based participatory research (CBPR) and other community‐engaged approaches; yet, few genomic/precision medicine studies include these community engagement strategies. Engagement can be challenging to implement due to the reliance of studies on large databases with pre‐existing biospecimen data and a lack of experience in engagement science among most genomic/precision medicine researchers.^8^, ^12^ The lack of racial/ethnic researcher workforce diversity is also evident in this field.^13^ Participants need to see themselves reflected in the composition of precision medicine research, not simply as token participants to fulfil enrolment goals but in leadership roles within the research team with legitimate decision‐making powers as well.

Authentic collaboration is sought from participants at the outset with meaningful influence in carrying out ‘checks and balances’ on responsible conduct of research. The capacity of any research program can be built by engaging participant stakeholders in collaborative decision‐making, facilitating dialogue, balancing power and disseminating new genetic information back to the participant community.^14^ Therefore, a new conceptual model for engagement is needed that focuses on the unique aspects of genomic/precision medicine that make engagement challenging. This paper endeavours to provide a framework using engagement science precepts to guide the involvement of groups underrepresented in research in priority‐setting, study design, implementation, study oversight, interpretation of results and dissemination of genomic/precision medicine research findings.

## 2 MATERIALS AND METHODS

We began our conceptual framework development by using the academic literature and expert consensus building to: (1) identify foundational concepts, (2) select a model of engagement to adapt, (3) identify themes unique to the engagement of racial/ethnic minorities in genomic/precision medicine and (4) adapt the model based on the themes. The key concepts included principles of community engagement^14^, ^15^, ^16^, ^17^ with diverse populations and the following:

1. While genomic variation is similar and mostly shared across populations and genomic diversity exists in clusters in populations with shared continental ancestry, there must be caution in assigning health‐related risks linked to polymorphisms within these groups to everyone with similar continental ancestry. The shared culture, experiences and environmental exposures may impact others outside the group with shared continental ancestry differently.^3^
2. Racial/ethnic minorities' willingness to participate in research is frequently shaped by cultural beliefs, social standing, personal and group experiences with health systems and research, discrimination and historical research abuses.
3. Factors exist influencing racial/ethnic minorities' trust specific to genomics, such as eugenics, fallacies about genetic inferiority, the history of exploitation of minorities in genomics and scientists' insensitivity to cultural concerns and ancestral beliefs.
4. Genomic/precision medicine researchers typically are not trained to identify and convene stakeholders, develop mutually beneficial partnerships with diverse communities, elicit feedback or integrate stakeholders into research.

Our transdisciplinary team comprised of individuals with substantial experience in Community‐Engaged Research (CEnR), health equity, precision medicine, biomedical ethics, engagement methodology and impact evaluation techniques proceeded to review existing conceptual models of CEnR to determine if any included the above concepts on diversity in genomic/precision medicine (Table 1, Search Strategy 1).

**Table 1:** Literature review search strategies

Database and Search Strategy 1	Search limits
*PubMed*	
Search (conceptual[Title]) AND (((((((community based participatory research[MeSH Terms]) OR engagement) OR community engaged) OR stakeholder) OR patient engagement) OR participatory))	Publication date from 1960/01/01 to 2017/12/31

Database and Search Strategy 2	Search limits
*PubMed*	
Search (((((((((((((((((((((((groups, minority[MeSH Terms]) OR minority health[MeSH Terms]) OR ethnic groups[MeSH Terms]) OR ethnicity[MeSH Terms]) OR diversity, genetic[MeSH Terms]) OR genetic diversity[MeSH Terms]) OR cultural diversity[MeSH Terms]) OR diversity) OR racial minority) OR ethnic minority) OR african continental ancestry group[MeSH Terms]) OR american native continental ancestry group[MeSH Terms]) OR ancestry group, oceanic[MeSH Terms]) OR asian continental ancestry group[MeSH Terms]) OR hispanic) OR latino) OR american indian[MeSH Terms]) OR african americans[MeSH Terms]) OR asian[MeSH Terms]) OR pacific islander american[MeSH Terms]) OR indigenous population[MeSH Terms]) OR australian aborigine[MeSH Terms])) AND ((((((genomics[MeSH Terms]) OR genetics[MeSH Terms]) OR precision medicine))) AND (((((((community based participatory research[MeSH Terms]) OR engagement) OR community engaged) OR stakeholder) OR patient engagement) OR participatory) OR patient centered care[MeSH Terms]))	Publication date from 1960/01/01 to 2017/12/31

As none of the 252 papers identified focused on genomic or precision medicine, we selected the conceptual model from Wallerstein and Duran,^15^ which has been widely applied to research with different minority groups.^16^ To adapt this existing model, we reviewed the literature to identify elements related to the four dimensions in the Wallerstein and Duran model: context, partnership processes and dynamics, intervention and research and outcomes (Table 1, Search Strategy 2). We used an iterative process to integrate and refine the new elements into the framework. Perspectives of our community partners were integrated into the adapted and final conceptual framework from various guided listening sessions held in diverse communities.

## 3 RESULTS

We identified seven themes unique to racial/ethnic minorities that may have an impact on involvement in genomic/precision medicine: (1) history of harm, exploitation and discrimination specific to genetics (e.g., claims of genetic inferiority); use of genomic and paleogenomic information in stigmatizing ways; (2) increased risk of group harm due to possible generalizations about groups related to genetics; (3) conflation of race and ethnicity (social constructs) with genetic ancestry; (4) perceived disproportionate risk of harm outside of medicine and health sector (e.g., use of DNA in the criminal justice system); (5) cultural and religious beliefs about ancestry and kinship; (6) disrespect of or failure to recognize tribal governance and (7) disparities in privacy risk when race, geolocation and social factors are included in data.^7^

### 3.1 Conceptual framework

**Figure 1:**
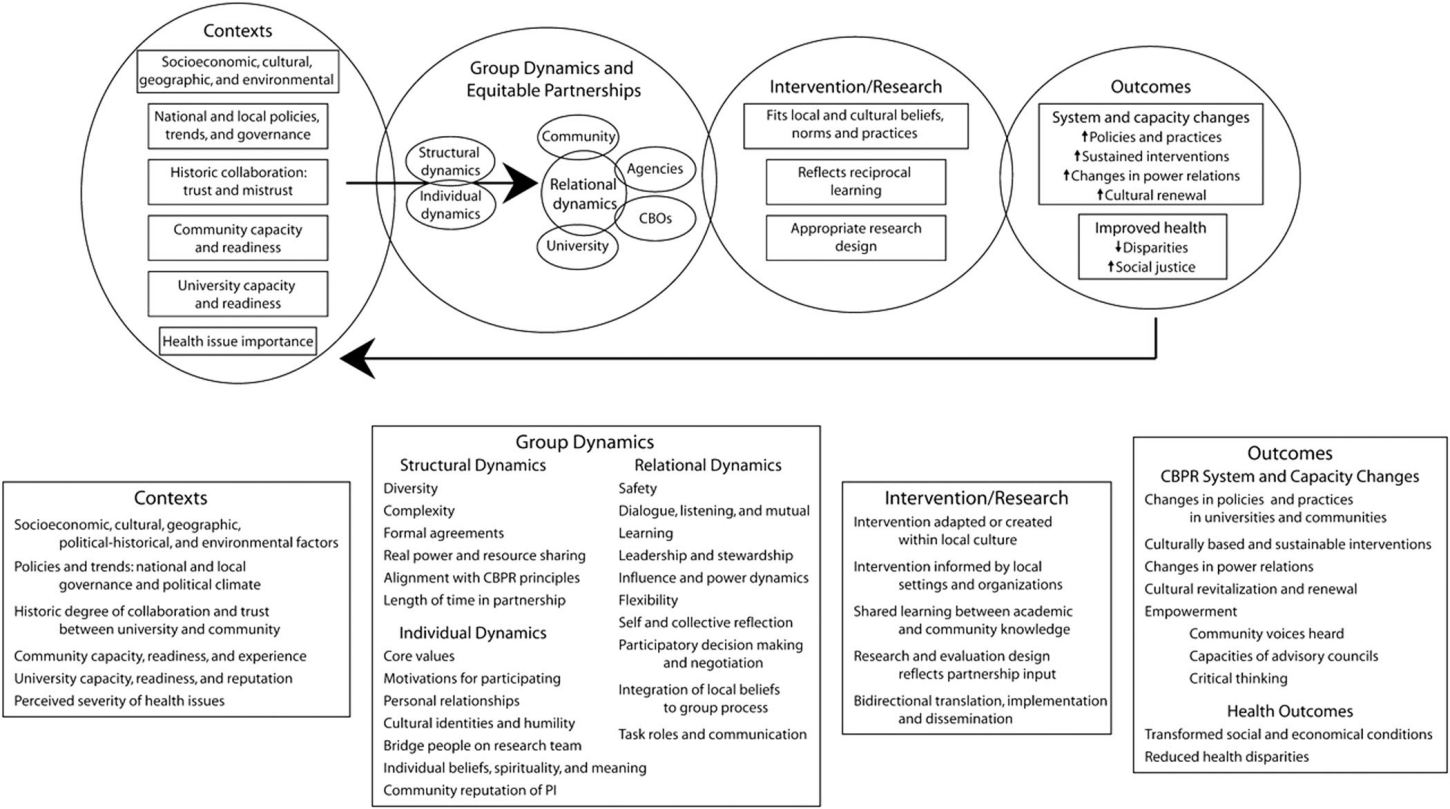
Conceptual Logic Model of community‐based participatory research^15^

**Figure 2:**
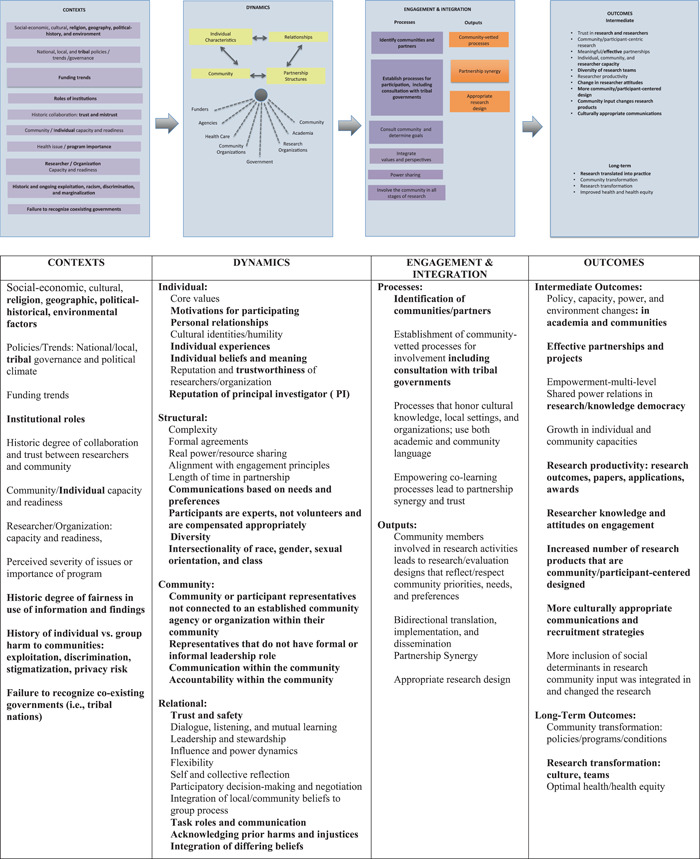
Engagement in precision medicine conceptual framework. Adapted from Wallerstein et al.^22^ and Wallerstein and Duran^15^

The seven themes above were used to review four overarching dimensions included in Wallerstein and Duran's^15^ conceptual model (Figure 1), which proposes that context grounds partnership processes, potentially impacting intervention and research, as well as outcomes. This model consists of specific theories or models, including community empowerment,^17^ person‐centeredness^18^ and individual dynamics.^19^ In the adapted model (Figure 2), changes and additions are bolded and italicized.

#### 3.1.1 First dimension: Context

The first dimension, ‘Context’, refers to macrolevel factors influencing engagement, including economic, social, cultural and spiritual contexts; environments; local/national policies; funding trends; institutional roles, history of trust/mistrust; and academic and community readiness to engage in research.^20^ We added the following context dimension: history of individual or group harm to communities, including exploitation, discrimination, privacy risk and stigmatization; and failure to recognize coexisting governments.

#### 3.1.2 Second dimension: Dynamics

The second dimension, ‘Dynamics’, includes the subdimensions of structural, individual, relational and community dynamics. Much of the literature on partnership processes is based on CBPR, where equitable partnerships are foundational. Relationship processes specific to precision medicine and genomic research are a less explored domain.

*Individual dynamics*: Individual‐level characteristics include core values, cultural identities, cultural humility and personal beliefs. We changed cultural identity to individual identity and included racial, ethnic, family and social identity. An individual may share beliefs and experiences with more than one group and explicitly name race and ethnicity as identities distinct from ancestry. Essentially, that race and ethnicity are social identities that are different from ancestry. We also added trustworthiness of the researcher/organization as an individual‐level perception. Since trustworthiness of the researcher/organization is an individual‐level perception as is the dynamic communities have with investigators as a factor in communities' decision to engage; reputation of the investigator was included in this dimension.

*Structural dynamics*: A structural marker of equality is valuing participants as experts and not ‘volunteers’, ensuring appropriate compensation commensurate with the participant's time. We expanded the concept of diversity to include place (rural vs. urban), race/ethnicity, gender identity and health status. We also added intersectionality, recognizing that social categories, such as race, class, gender and sexual orientation intersect and amplify the dynamics of privilege and oppression.^21^ These factors are critical to understanding gene–environment interactions and capturing the range of human experiences including lifestyle, which is key in precision medicine.

*Relational dynamics*: Trust is at the crux of relational dynamics as a fluid relational characteristic, earned by experience, follow‐through and fairness. To engender this dynamic is to create an environment of dialogue and cocreation. Intentionality should be applied to diffuse power dynamics by providing clear expectations and resources for full participant engagement. We added acknowledging prior harms/injustices and integration of differing beliefs.

*Community dynamics:* We included the new subdimension to represent community or participant representatives not connected to a community or an organization. These representatives are not currently accounted for in national studies and differ from those in CBPR, where representatives usually have some leadership role in the community and are viewed as key informant leaders.

#### 3.1.3 Third dimension: Engagement and integration

We changed the third dimension from ‘Intervention and Research’ to ‘Engagement and Integration’ to be more reflective of activities in engaged genomic/precision medicine. For example, in CBPR, communities tend to have a clear history of geographical and/or cultural connectedness, while in genomic/precision medicine, individuals may share similar beliefs and experiences but are geographically dispersed. Investigators may benefit from engaging and managing power imbalances that arise in the context of engaged work. These might include modelling humility, having self‐awareness, allowing space for the voices of community members and valuing the lived experience and indigenous knowledge (both in the community and in the study investigators) that community members bring. These modalities can be employed using remote technologies via web conference platforms*.* We distinguished engagement from recruitment in this dimension, emphasizing that engagement is bidirectional and fully involves participants in research. Engagement should begin with the establishment of community‐vetted processes, including consultation with tribal governments, when appropriate. Other aspects of engagement include integration of communities' knowledge, priorities and concerns to create community and participant‐centred programmes; strategies facilitating partnership synergy, promoting colearning and considering the resources required for communities to engage; development of policies, education and training (for both community members and researchers) and integration of community viewpoints in the design, conduct, dissemination and evaluation of research.^23^, ^24^

#### 3.1.4 Fourth dimension: Outcomes

The ongoing interaction between the context, partnering processes and implementation of engagement in research leads to the fourth dimension: outcomes. Outcomes range from intermediate systems to more distal ‘long‐term’ outcomes, such as redefining research ‘subjects’ as ‘participants’, changing researcher's attitudes and shifting to a more participant‐centred consent process.

We added transformation of research, the inclusion of diverse research teams and innovative research approaches based on models developed jointly by community and academic partners. Research transformation is apparent when community input is integrated into research approaches, changing research products to include more community‐/participant‐centred designs, resulting in culturally appropriate engagement and recruitment strategies.

## 4 DISCUSSION

The growing volume of genomic/precision medicine research studies requires the engagement of diverse stakeholders to ensure the most precise assessment of genetic, social and environmental factors. Our conceptual framework considers barriers experienced by racial/ethnic minorities in genomic/precision medicine and integrates key themes related to the history of harm and increased privacy risk of minority groups. We differentiate engagement and recruitment while addressing ethical, legal and social issues in genomic/precision medicine.

Similar to the original model, this revised framework is a dynamic entity with four dimensions that allow research teams the flexibility to identify contexts, dynamics, engagement and outcomes specific to their work. We recommend applying this framework to:

1. Engage participants in consciousness‐raising about genomics, fostering their needed input on research priorities;
2. Mitigate barriers to involving participants in genomic research through governance, oversight and decision‐making, effectively eliminating structural disadvantages;
3. Encourage genomic scientists to forge partnerships with underrepresented communities to ethically map genomic research priorities and ensure equipoise in dissemination;
4. Engage early with the community before the design stage of research and encourage various forms of participant input (i.e., targeted questions and short turnaround time or long‐term input that endures over time).

Instruments and methods that reliably assess and measure the impact of engagement beyond research participation are fundamentally required. Quantitative approaches that incorporate rigorous mixed‐methods evaluation and employ evidence‐based models of best practice in engagement are advised. A comprehensive evaluation plan focusing on process and outcome benchmarks may shed light on strengths and weaknesses in engaging participants and communities bidirectionally in the research enterprise.

## 5 CONCLUSION

Involving participants and communities in genomic/precision medicine research is a complex process. Intentionally coordinated participant engagement will foster a more participant‐centric research approach, potentially producing more culturally relevant tools, increased trust and policies facilitating citizen science. Our adapted framework addresses some of the existing gaps among best‐practice tools for participant engagement in ‘Genomic/Precision Medicine’ and recommends applied approaches with research participants as consumers in the genomics enterprise. The framework is not intended to link the community with genomic/precision medicine. The purpose is to guide the engagement of communities in research that focuses on genomic/precision medicine*.* This conceptual framework is well‐suited for further modification to pair implementation strategies and evaluation tools to its processes and outcome measures. This study highlights the need for further efforts to examine the impact, effectiveness and scalability of our framework to future testable models.

## AUTHOR CONTRIBUTIONS

Consuelo H. Wilkins conceived the study, and all authors identified key literature to be included in the review. All of the authors participated in the development and refinement of the conceptual framework, including discussions and drawing on the community partner's perspectives, with leadership from Consuelo H. Wilkins and Karriem S. Watson. Alecia M. Fair and Selena M. Carpenter managed the references and design of the figure and table. Karriem S. Watson led the drafting of the manuscript and key discussion points, with subsequent versions modified and manuscript preparation by Alecia M. Fair, Selena M. Carpenter, Consuelo H. Wilkins, Elizabeth G. Cohn, Karriem S. Watson, Laura A. Szalacha and Usha Menon. All authors provided important intellectual contributions with guidance from Consuelo H. Wilkins throughout the development of the manuscript, final revisions and presentation of the conceptual framework. All the authors contributed to critical review and revisions to the manuscript, agreeing on the final version.

## CONFLICTS OF INTEREST

The authors declare no conflicts of interest.

## Data Availability

Data sharing is not applicable to this article as no data were created or analyzed in this study.
